# Case report: atypical presentation of mpox with massive hematochezia and prolonged viral shedding despite tecovirimat treatment

**DOI:** 10.1186/s12879-024-09098-2

**Published:** 2024-02-12

**Authors:** Sung Un Shin, Younggon Jung, Seong Eun Kim, Dong Min Kim

**Affiliations:** 1 Department of Internal Medicine, Chonnam National University Medical School, Gwangju, South Korea; 2 Department of Internal Medicine, St. Carollo Hospital, Suncheon, South Korea; 3 Department of Internal Medicine, College of Medicine, Chosun University, Gwangju, South Korea

**Keywords:** Mpox, Rectal ulceration, Isolation, Hematochezia, Bleeding, Sexual transmitted infections (viral)

## Abstract

**Background:**

The outbreak of mpox that occurred between 2022 and 2023 is primarily being transmitted through sexual contact. As of now, there is no consensus on the recommended duration of isolation to prevent sexual transmission of the virus. Moreover, this particular mpox outbreak has presented with distinct complications in comparison to previous occurrences. In this report, we present a case involving severe rectal bleeding from an ulcer in a mpox patient with a history of engaging in receptive sexual contact.

**Case presentation:**

A 30-year-old Korean man presented at the hospital with complaints of fever, multiple skin lesions, and anal pain. Monkeypox virus polymerase chain reaction (PCR) results were positive for skin lesions on the penis and wrist. The patient received a 12-day course of tecovirimat due to anal symptoms and perianal skin lesions. Following isolation for 12 days and after all skin scabs had naturally fallen off, with no new skin lesions emerging for a consecutive 48 hours—conforming to the criteria of the Korean Disease Control and Prevention Agency—the patient was discharged. However, 1 day after discharge, the patient returned to the hospital due to hematochezia. His hemoglobin level had significantly dropped from 14.0 g/dL to 8.2 g/dL. Sigmoidoscopy unveiled a sizable rectal ulceration with exposed blood vessels, prompting the application of hemostasis through metal clipping. Subsequent monkeypox virus real-time PCR conducted on rectal tissue and swabs yielded positive results (with cycle threshold values of 28.48 and 31.23, respectively). An abdominal CT scan exposed a perirectal abscess, for which ampicillin-sulbactam was administered.

**Conclusion:**

This case underscores the importance of monitoring for bleeding complications and confirming the resolution of rectal lesions before discharging patients from isolation, particularly in cases where patients have a history of engaging in receptive sexual contact with men or are presenting with anal symptoms.

## Background

Mpox (formerly denominated monkeypox) is a zoonotic disease that caused by monkeypox virus of the Poxviridae family and Orthopoxvirus genus. Its human transmission was first recognized in 1970 and mpox predominantly manifested as an endemic disease in Central and West Africa until the 2022 outbreaks. The outbreak reached a turning point in May 2022 when Europe recorded its first mpox case. This incident was quickly followed by an uptick in cases from countries previously considered non-endemic. As of August 18, 2023, the outbreak, characterized by a surge in cases among young gay, bisexual, and other men who have sex with men (GBMSM), had registered 89,385 confirmed infections [1]. Although isolation in healthcare settings is widely recommended to prevent transmission of mpox, guidelines generally advocate for isolation until the patient’s scabs have fallen off and no new lesions appear, thereby preventing direct skin-to-skin transmission. However, the 2022–2023 mpox outbreak is primarily transmitted through sexual contact, and to date, there is no consensus on the duration of isolation to prevent sexual transmission especially in the GBMSM population, where mpox is primarily transmitted. In addition, the 2022–2023 monkeypox outbreak is presenting with distinct complications compared to previous outbreaks [2].

We report a case of major bleeding from rectal ulcer in men who have sex with men (MSM) with a history of receptive sexual contact and demonstrate that monkeypox virus DNA shedding from rectal tissue can occur for a longer period even after the skin lesions were all healed.

## Case presentation

A 30-year-old male living in South Korea visited a secondary general hospital (hospital A, as follows), complaining of multiple skin lesions that had started 1 week prior. He was also experiencing lower abdominal discomfort and a yellowish discharge from the anus that had begun 2 days prior. The skin lesions were present throughout his body, including the hands, feet, and anogenital area. Inguinal tender lymphadenopathy was also noted during the physical examination. The skin lesions consisted of ulcers, umbilicated pustules, and erythematous plaques with central erosion. He had no travel history to mpox endemic countries, such as Central or West Africa, nor to recent outbreak countries in Europe or the United States within 3 weeks before symptom onset. He reported having had receptive sexual intercourse with an anonymous male partner 1 week before the symptoms initiated. A polymerase chain reaction (PCR) test result was positive, with a cycle threshold (Ct) value of 24.23, obtained from a penile ulcer and left wrist pustules.

**Fig. 1:**
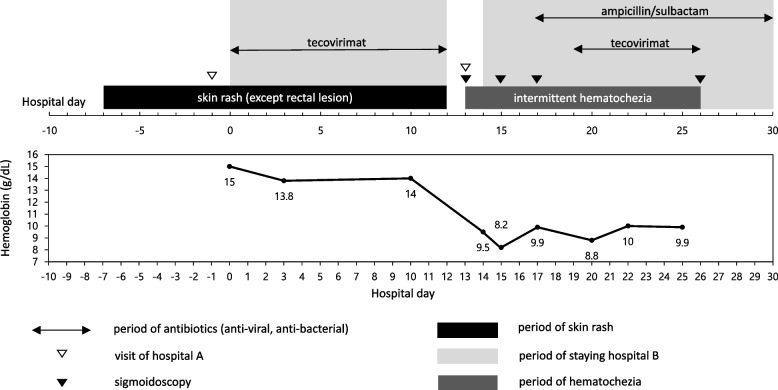
Clinical course of the patient

**Fig. 2:**
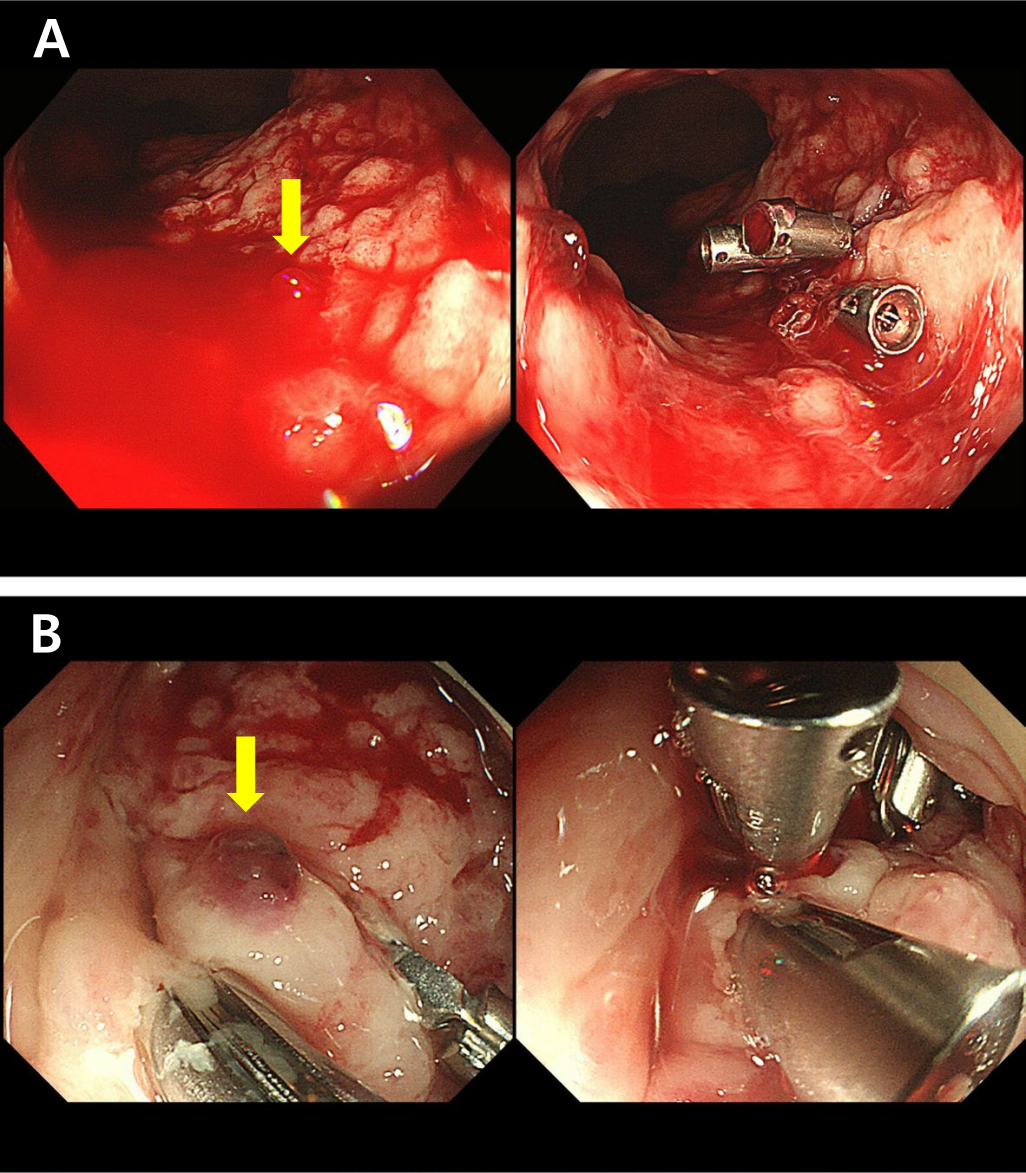
(**A**) First sigmoidoscopy on hospital day 13 (**B**) Third sigmoidoscopy on hospital day 17

**Fig. 3:**
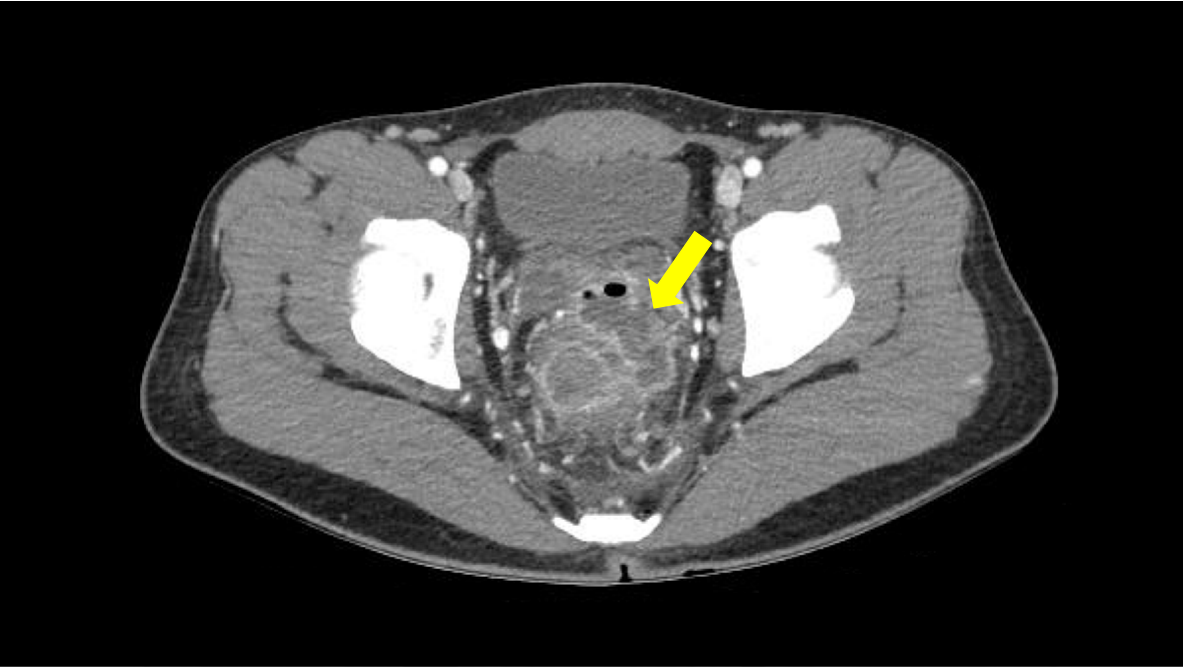
Abdominal computed tomography on hospital day 15

He was transferred to a university-affiliated tertiary hospital (hospital B, as follows) for isolation and treatment for mpox and this is Hospital Day (HD) 0. Some of the skin lesions had formed scabs at the time of hospital admission. Human Immunodeficiency Virus (HIV) antibody testing was negative, and all routine laboratory tests were normal except for an elevated C-reactive protein level of 6.59 mg/dL (normal range: 0–0.3 mg/dL). Tecovirimat was administered orally at a dose of 600 mg every 12 hours due to the infection site being the anal area, and because he complained of anal pain. On HD6, scabs formed on most skin lesions, and the anal pain improved. On HD8, scabs formed on skin lesions, and by HD11 all skin lesions had lost their scabs. After ensuring the absence of new lesions for over 48 hours, the patient was discharged on HD12. Tecovirimat was administered for 12 days during the first hospitalization. A diagram of the patient’s clinical course is shown in Fig. 1. One day after discharge (HD13), the patient experienced massive hematochezia and revisited the emergency room of hospital A which was he had initially visited when his symptoms appeared. On sigmoidoscopy, spurting bleeding was observed 3 cm above the anus, and hemostatic clipping was performed on the lesion (Fig. 2A). He was transferred to hospital B after initial hemostasis for further treatment. After transfer, real-time PCR tests were conducted for both monkeypox virus and herpes virus using rectal swab specimens (HD15) as well as rectal tissue obtained through follow-up colonoscopy (Fig. 2B, HD17). The results were positive for monkeypox virus with Ct values of 31.23 and 28.48, respectively, but negative for herpes simplex virus 1 and 2. From HD15 to HD26, the patient presented with intermittent episodes of hematochezia, and the lowest hemoglobin level recorded was 8.2 g/dL. Abdominal angiographic computed tomography (CT) on HD15 showed proctocolitis and a perirectal abscess without contrast extravasation (Fig. 3). The perirectal abscess size was 4.8 cm × 1.6 cm. Intravenous administration of ampicillin/sulbactam was initiated from HD16 to HD28. Same dose of tecovirimat was reintroduced for 7 days after a positive monkeypox virus PCR result was reported from the rectal tissue.

After HD26, the patient did not experience any additional signs of bleeding, such as hematochezia or decreasing hemoglobin levels. Follow-up sigmoidoscopy and abdominal CT showed improvement in the several linear ulcers in the distal rectum and a decreased abscess size compared to the previous examination on HD26 and HD27. Antibiotics were switched to oral amoxicillin/sulbactam from HD28, and the patient was discharged on HD30.

## Discussion and conclusions

The 2022–23 mpox outbreak has shown different complications compared to previous outbreaks. Earlier outbreaks primarily involved secondary bacterial skin infections or pneumonia, whereas recent reports indicate rectal pain, proctitis, penile edema, and tonsillar or pharyngeal ulceration with associated swallowing difficulties [2]. This increase in unique complications is thought to be associated with transmission of the virus through direct sexual contact among MSM. Recent studies have reported proctitis in mpox patients ranging from 14 to 31% [3, 4]. Another study reported a significantly higher rate of proctitis in MSM who engage in anal receptive sex compared to MSM who do not engage in anal receptive sex (38% vs. 7%) [5]. It can be inferred that MSM history of receptive sex is associated with the development of proctitis in this patient. In addition to proctitis, rectal bleeding has been reported in 4–11% of mpox patients [6, 7]. However, we could not find any reports of major bleeding causing a decrease in hemoglobin as seen in present case. This case demonstrates that major bleeding can occur in mpox patients with rectal ulcer.

Previously, mpox was known to be transmitted through direct skin-to-skin contact, but a study of epidemiologic and clinical data from the 2022 mpox outbreak confirmed that sexual contact is more effective than casual skin-to-skin contact [8]. To prevent transmission through sexual contact, it is necessary to determine the duration for which viral shedding occurs in the semen, rectum, and vagina. In a previous study, mpox viral DNA was detectable by quantitative PCR for a median time of 16 days (IQR 13–23 days) in the rectum from symptom onset in immunocompetent mild mpox patients [4]. And there is a study suggested that a Ct value of ≥ 35 corresponds with non or marginal infectivity [9]. In another study, a significant difference was observed between the median Ct value of samples that grew in viral culture (Ct 22; range 16–36) compared to samples that did not grow (Ct 33; range 26–40) (*p* < 0.001) [10]. In present case, the Ct value of rectal tissue monkeypox real time PCR was 28.48 on day 24 from symptom onset, suggesting prolonged viral presence compared to usual mpox patients. This suggests that severe complicated mpox patient with rectal ulcer could have prolonged viral shedding and longer isolation period should be considered.

In 2018, the U.S. FDA approved tecovirimat for the treatment of smallpox in children and adults, based on its efficacy in rabbitpox infection in non-human primates. However, the safety and efficacy of tecovirimat in mpox patients have not been established. In the present case, despite the use of tecovirimat for 12 days from the first hospitalization, worsening of the rectal lesion and bleeding occurred, and a positive rectal tissue monkeypox PCR result was confirmed on the second hospitalization. This case suggests a low efficacy of tecovirimat treatment in patients with mpox. This case suggests the need for further clinical studies on the efficacy of tecovirimat in patients with mpox.

This case report illustrates the severe complications, including major rectal bleeding, that can arise in patients with mpox, particularly among those with a history of receptive sexual contact. The prolonged viral shedding observed in our patient, despite treatment with tecovirimat, underscores the potential for persistent infectivity. This raises significant questions regarding the optimal duration of patient isolation and the effectiveness of tecovirimat treatment for mpox. Given the unpredictability and severity of the clinical course of mpox, as evidenced by this patient, vigilant monitoring is paramount, even after the resolution of skin lesions and after isolation termination. This case strongly advocates for the development of rigorous protocols for the evaluation of mucosal and internal lesions, in addition to skin lesions, and for the management of potential bleeding complications, particularly in patients with a history of receptive sexual contact. Further research is necessary to identify the most effective treatment strategies for mpox, to delineate the natural history of viral shedding in various body compartments, and to refine isolation criteria to reduce the risk of ongoing transmission and complications.

## Data Availability

Data sharing is not applicable to this article as no datasets were generated or analysed during the current study.

## Abbreviations

PCR: Polymerase chain reaction
GBMSM: Gay, bisexual, and other men who have sex with men
Ct: Cycle threshold
HIV: Human immunodeficiency virus
HD: Hospital day
CT: Computed tomography
MSM: Men who have sex with men
IQR: Interquartile range
FDA: Food and drug administration
